# α-thalassaemia

**DOI:** 10.1186/1750-1172-5-13

**Published:** 2010-05-28

**Authors:** Cornelis L Harteveld, Douglas R Higgs

**Affiliations:** 1 Department of Human and Clinical Genetics, Leiden University Medical Center, Einthovenweg 20, 2333ZC Leiden, The Netherlands; 2 Medical Research Council Molecular Haematology Unit, Weatherall Institute of Molecular Medicine, The John Radcliffe Hospital, Headington, Oxford, OX3 9DS, UK

## Abstract

Alpha-thalassaemia is inherited as an autosomal recessive disorder characterised by a microcytic hypochromic anaemia, and a clinical phenotype varying from almost asymptomatic to a lethal haemolytic anaemia.

It is probably the most common monogenic gene disorder in the world and is especially frequent in Mediterranean countries, South-East Asia, Africa, the Middle East and in the Indian subcontinent. During the last few decades the incidence of alpha thalassaemia in North-European countries and Northern America has increased because of demographic changes. Compound heterozygotes and some homozygotes have a moderate to severe form of alpha thalassaemia called HbH disease. Hb Bart's hydrops foetalis is a lethal form in which no alpha-globin is synthesized. Alpha thalassaemia most frequently results from deletion of one or both alpha genes from the chromosome and can be classified according to its genotype/phenotype correlation. The normal complement of four functional alpha-globin genes may be decreased by 1, 2, 3 or all 4 copies of the genes, explaining the clinical variation and increasing severity of the disease. All affected individuals have a variable degree of anaemia (low Hb), reduced mean corpuscular haemoglobin (MCH/pg), reduced mean corpuscular volume (MCV/fl) and a normal/slightly reduced level of HbA_2_. Molecular analysis is usually required to confirm the haematological observations (especially in silent alpha-thalassaemia and alpha-thalassaemia trait). The predominant features in HbH disease are anaemia with variable amounts of HbH (0.8-40%). The type of mutation influences the clinical severity of HbH disease. The distinguishing features of the haemoglobin Bart's hydrops foetalis syndrome are the presence of Hb Bart's and the total absence of HbF. The mode of transmission of alpha thalassaemia is autosomal recessive. Genetic counselling is offered to couples at risk for HbH disease or haemoglobin Bart's Hydrops Foetalis Syndrome. Carriers of alpha^+^- or alpha^0^-thalassaemia alleles generally do not need treatment. HbH patients may require intermittent transfusion therapy especially during intercurrent illness. Most pregnancies in which the foetus is known to have the haemoglobin Bart's hydrops foetalis syndrome are terminated due to the increased risk of both maternal and foetal morbidity.

## Introduction

Why should α thalassaemia be considered in a forum dedicated to rare diseases? It is certainly not a rare genetic trait. On the contrary, it is one of the most common human genetic abnormalities known. Carriers of α thalassaemia are found at polymorphic frequencies (>1%) in all tropical and subtropical populations that have been studied and, in some areas, the carrier state has almost gone to fixation. This is because carriers of α thalassaemia are thought to be at a selective advantage in areas where falciparum malaria is or has been endemic. In areas where the carrier state is common, two clinically important diseases (HbH disease and Hb Bart's hydrops foetalis) occur in compound heterozygotes and homozygotes. The reason for discussing this here is therefore not because these diseases are rare, rather that they may be rarely considered by physicians outside of the regions where thalassaemia commonly occurs. For example, a retrospective study of obstetric records in the U.K. by Petrou et al. revealed an underdiagnosis of both α^0^-thalassaemia trait and α-thalassaemia hydrops foetalis[1]. With the massive migrations that have occurred over the past few decades it is important to bring these rarely considered diseases to the general attention of clinicians in Northern Europe and North America.

## Disease names and synonyms

The generic term α thalassaemia encompasses all of those conditions in which there is a deficit in the production of the α globin chains of haemoglobin (Hb) which is a tetrameric molecule including two α-like and two β-like globin chains (α_2_β_2_). Underproduction of α globin chains gives rise to excess β-like globin chains which form γ_4 _tetramers, called Hb Bart's (in foetal life) and β_4 _tetramers, called HbH (in adult life). Individuals who carry mutations affecting the α globin genes on one chromosome, associated with minimal anaemia, are said to have α thalassaemia trait. Compound heterozygotes and some homozygotes for α thalassaemia have a moderately severe anaemia characterised by the presence of HbH in the peripheral blood. This condition is referred to as HbH disease. Finally some individuals who make very little or no α globin chains have a very severe form of anaemia which, if untreated, causes death in the neonatal period. This condition is called the Hb Bart's hydrops foetalis syndrome [2-5].

Rarely patients have been seen with very large deletions which remove the α globin genes but also remove many other genes that surround them. This condition is associated with developmental abnormalities (including intellectual disability) and is referred to as the α thalassaemia/mental retardation syndrome on chromosome 16 (ATR16 syndrome: OMIM:141750, reviewed in Higgs et al., 2009 [6] and Wilkie et al., 1990 [7]). Also patients with a rare form of syndromal X-linked mental retardation associated with α thalassaemia have been described, in which the intellectual disability is more severe and the dysmorphic features show striking similarities among patients. This rare condition is called ATR-X syndrome and has been found to involve mutations in a chromatin associated protein called ATRX on the X-chromosome (ATR-X syndrome: OMIM:301040, reviewed elsewhere)[6,8-11]. Finally, an acquired form of alpha-thalassaemia referred to as the ATMDS syndrome has been described. This predominantly occurs in elderly males with a pre-malignant, clonal haematopoietic disease called myelodysplasia (MDS). This rare syndrome involves acquired mutations in the ATRX gene causing α thalassaemia (OMIM:300448, reviewed in Gibbons et al., 2003;Higgs et al., 2009)[6,12]. Since these rare conditions have all been reviewed elsewhere they will not be discussed further in this synopsis.

## Definition/Diagnostic Criteria

Alpha thalassaemia is most frequently suspected initially on the basis of a routine full blood count. All affected individuals have a variable degree of anaemia (Hb), reduced mean corpuscular haemoglobin (MCH/pg), reduced mean corpuscular volume (MCV/fl) and a normal or slightly reduced level of the minor HbA_2_. These parameters are discussed in greater detail below. When the level of α globin synthesis falls below ~70% of normal, in the foetal period, excess γ globin chains form Hb Bart's which can be detected on routine Hb analysis [13-19]. In adult life, excess β globin chains form β_4 _tetramers of HbH in the cell and these can be identified by staining the peripheral blood with 1% brilliant cresyl blue (BCB)[20-22], or when present in sufficient quantity by routine Hb analysis[20,23]. Previously α thalassaemia was confirmed by globin chain biosynthesis, when the α/β globin chain biosynthesis ratio was reduced to less than ~0.8[24-28]. All of these parameters are reduced in α thalassaemia but none of them alone or in combination can accurately or consistently predict the genotype for which directed molecular analysis of the α globin cluster is required and this is discussed below.

## Epidemiology

**Figure 1:**
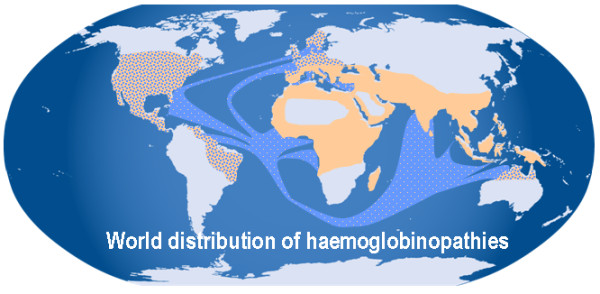
**The world distribution of haemoglobinopathies overlaps the geographic distribution of malaria**. The prevalence has increased in previously non-endemic areas as a consequence of historical and recent immigration flows, slave-trade, trading activities and colonization. In all these regions there is a high prevalence of a thalassaemia. It is believed that carriers of α thalassaemia are protected against malaria and that natural selection is responsible for elevating and maintaining their gene frequencies.

Like all common globin gene disorders (sickle cell trait and β thalassaemia) α thalassaemia occurs at high frequencies throughout all tropical and subtropical regions of the world (Figure 1). In some areas, the carrier frequency of α thalassaemia may be as high as 80-90% of the population, almost at fixation[29-33]. It is thought that all of these globin gene disorders (including α thalassaemia) have been selected because in some way they protect carriers from the ravages of falciparum malaria. The micro epidemiological evidence supporting this is very strong[34,35]. The mechanisms underlying this protection have been extensively studied but remain unknown. Of all globin disorders, α thalassaemia is the most widely distributed and therefore many individuals in these areas have interacting combinations of these variants (e.g. both α and β thalassaemia). Due to differences in the interactions between the various molecular defects underlying α thalassaemia (see below) HbH disease is predominantly seen in South East Asia, the Middle East and the Mediterranean. Similarly the Hb Bart's Hydrops foetalis syndrome is predominantly seen in South East Asia[36-41]. In passing it should be mentioned that ATR16, ATR-X and ATMDS syndromes show no geographical bias in their distributions.

Although the previously established distribution of α thalassaemia is represented in Figure 1, over the past few decades there have been massive population movements so that now the globin gene disorders, thought to be rarities in North European and North American clinical practice, have become major diagnostic and therapeutic challenges for our current health care systems[42].

## Clinical description

The clinical phenotypes of most individuals with α thalassaemia are very mild and may not be noticed during life other than when a routine full blood count is examined. Patients with HbH disease have a variable phenotype and those with Hb Bart's hydrops foetalis have a lethal form of anaemia.

### α Thalassaemia trait

Apart from mild to moderate microcytic hypochromic anaemia (detected on a routine blood count), carriers (heterozygotes) of α thalassaemia, whatever the molecular basis (see below), are clinically asymptomatic and the diagnosis (when made) is often established during a regular health check or during antenatal screening. Complaints related to more severe anaemias, such as fatigue, listlessness and shortness of breath are uncommon and almost certainly related to other concomitant disorders.

### HbH disease

HbH disease is most frequently seen in patients who are compound heterozygotes for two different mutations or less frequently homozygotes for a moderately severe molecular defect. They usually produce less than 30% of the normal amount of α globin. The predominant features in HbH disease are anaemia (2.6-13.3 g/dl) with variable amounts of HbH (0.8-40%), occasionally accompanied by Hb Bart's in the peripheral blood. The patients usually have splenomegaly (which may be severe) and occasionally this is complicated by hypersplenism. Jaundice may be present in variable degrees and children may show growth retardation. Other complications include infections, leg ulcers, gall stones, folic acid deficiency and acute haemolytic episodes in response to drugs and infections[5,43]. Older patients often have some degree of iron overload. The severity of the clinical features is clearly related to the molecular basis of the disease[5,43,44]. Patients with non-deletional types of HbH disease are more severely affected than those with the common deletional types of HbH disease[45-53].

### Hb Bart's Hydrops Foetalis Syndrome

**Figure 2:**
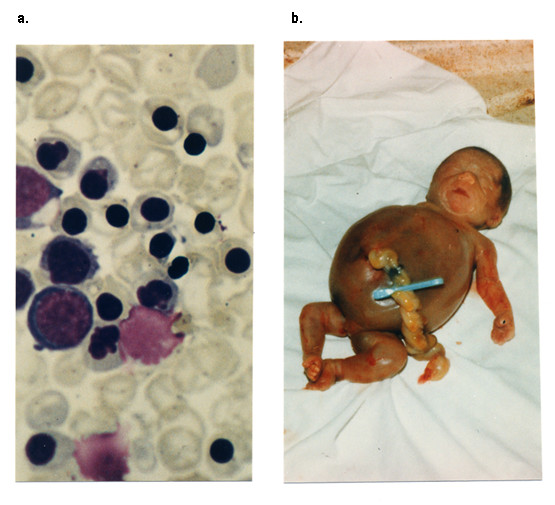
**The Haemoglobin Bart's hydrops syndrome**. a. peripheral blood film with immature red-cell precursors and hypochromic, microcytic, red cells showing anisocytosis and poikilocytosis; b. stillborn hydropic infant [5].

Infants with the Hb Bart's hydrops foetalis syndrome have the most severe deficiencies in α globin expression. While it most frequently results from the inheritance of no α globin genes from either parent, in some cases it results from the inheritance of a severe nondeletion mutation from one parent and no α genes from the other. Patients on the borderline between severe HbH disease and Hb Bart's hydrops foetalis syndrome are said to have HbH hydrops syndrome [45,52,54-56]. Physiologically non-functional homotetramers γ_4 _and β_4 _make up most of the haemoglobin in the erythrocytes in infants with the Bart's hydrops foetalis syndrome. They also have variable amounts of an embryonic Hb Portland (ζ_2_γ_2_), which is the only functional Hb in these infants and must be the only oxygen carrier keeping these infants alive. The clinical features are those of a pale oedematous infant with signs of cardiac failure and prolonged intra-uterine anaemia (Figure 2). Pronounced hepatosplenomegaly, retardation in brain growth, skeletal and cardiovascular deformities and gross enlargement of the placenta are characteristic features. Infants with the Hb Bart's hydrops foetalis syndrome almost always either die *in utero *(23-38 weeks) or shortly after birth, although few cases have been described in which the neonate is given intensive life-support therapy and treated with blood transfusion [57-60].

## Aetiology (Molecular Basis)

**Figure 3:**
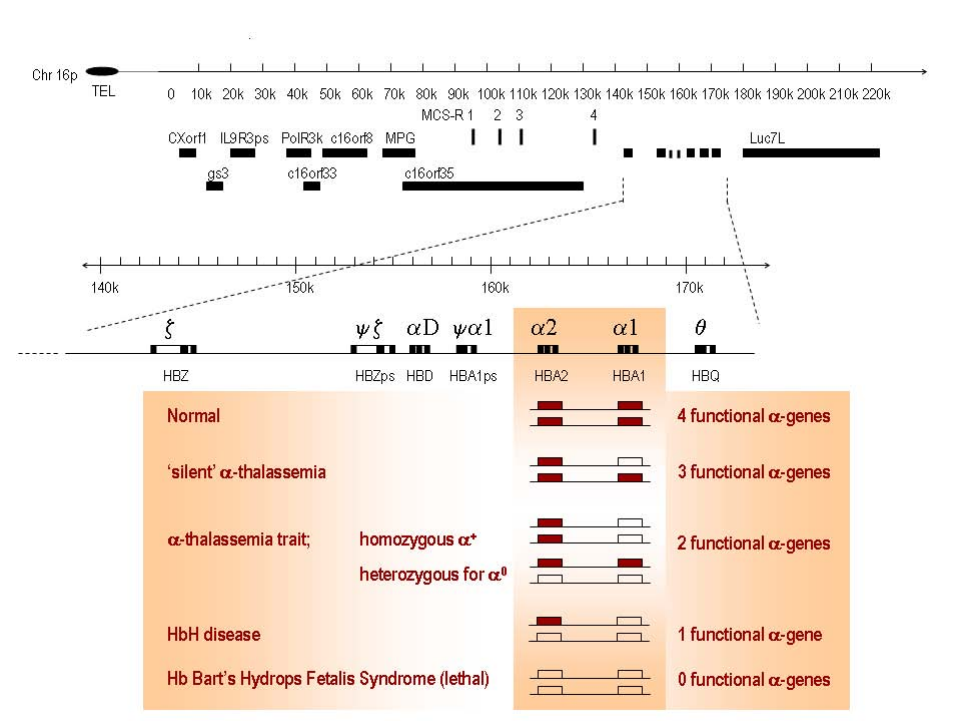
**The structure of the α-globin gene cluster on chromosome 16**. The telomere is shown as an oval, genes in the region are shown as boxes. The α-globin regulatory region (MCS-R 1 to 4) is indicated as vertical bars. The scale is in kilobases as indicated above. The alpha-gene cluster is enlarged showing the traditional gene names above and the HGVS gene names below. The table below shows the classification of gene defects and phenotypic expression.

In normal individuals α globin synthesis is regulated by four α globin genes two on each copy of chromosome 16 (in band 16p13.3 Figure 3) and this genotype is written as αα/αα. Expression of these genes is dependent on remote regulatory elements (named Multispecies Conserved Sequences or MCS-R1 to R4) located far upstream of the α globin genes in the introns of a flanking, widely expressed gene (Figure 3). Alpha thalassaemia most frequently results from deletion of one (-α) or both (--) α genes from the chromosome. Occasionally point mutations in critical regions of the α2 (α^T^α) or α1 (αα^T^) genes may cause, so-called, nondeletional α thalassaemia. Very rarely, α thalassaemia results from deletion of the MCS-R regulatory elements (written as (αα)^T^), in all of these deletions MCS-R2 is always removed and thus appears to be the major regulatory element. When a mutation(s) completely abolishes expression from a chromosome this is called α^0^-thalassaemia and when the mutation(s) only partially downregulate expression from the chromosome this is called α^+^-thalassaemia.

### α^+^-thalassaemia due to deletions

**Figure 4:**
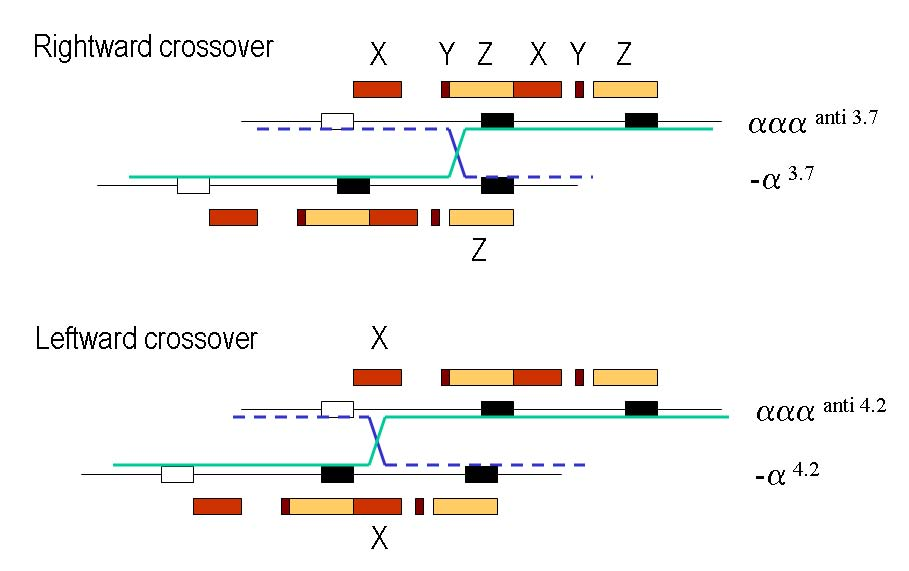
**Deletions that cause α^+^-thalassaemia**. The homologous duplication units X, Y and Z in which the α-genes are embedded are indicated as colored boxes. A cross-over between the mis-paired Z boxes during meiosis gives rise to the -α^3.7 ^and ααα^anti 3.7 ^chromosomes. Cross-over between misaligned X-boxes give rise to -α^4.2 ^and ααα^anti 4.2^.

**Figure 5:**
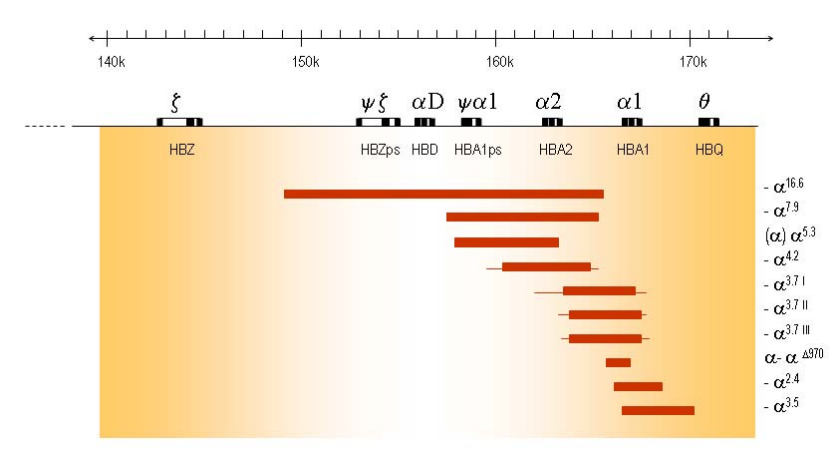
**Deletions of one α-gene giving rise to α^+^-thalassaemia**. The extent of the deletion is shown as bars, thin lines indicate regions of uncertainty of the breakpoints.

The α-globin genes are embedded within two highly homologous 4 kb duplication units [61-65]. One very common α-thalassaemia deletion is the rightward deletion, a 3.7 kb deletion caused by reciprocal recombination between Z segments producing a chromosome with only one functional α-gene (α-^3.7 ^or rightward deletion) causing α-thalassaemia and an α-triplication allele without a thalassaemic effect (Figure 4). Likewise a reciprocal recombination between mispaired X-boxes results in a 4.2 kb deletion, called leftward deletion (-α^4.2^) [61,66-68]. An increasing number of deletions resulting in the loss of a single α-gene are reported due to non-homologous recombination events, most of which are rare, or highly region specific. The most common α^+^-thalassaemia deletions are shown in Figure 5. More extensive overviews of all deletions are reported elsewhere (in: Disorders of Hemoglobin Cambridge University Press 2009) [69,70].

### α^+^-thalassaemia due to non-deletion types of α-thalassaemia

Alpha-thalassaemia is more frequently caused by deletion than single point mutations or nucleotide insertions and deletions involving the canonical sequences controlling gene expression. In general the non-deletion α^+^-thalassaemia determinants may give rise to a more severe reduction in α-chain synthesis than the -α deletion type of chromosomes. Many mutations have been described affecting mRNA processing, mRNA translation, and α-globin stability. Table 1 shows all the currently known non-deletion mutants causing α^+^-thalassaemia. Of these the most common non-deletional variants are the α^IVSI(-5 nt)^α (in Mediterraneans), polyadenylation site mutations α_2_^AATAAG^, α_2_^AATGAA ^and α_2_^AATA-- ^(in the Mediterranean and Middle East)[71-74], termination codon mutations leading to elongated Hb variants, such as Hb Constant Spring (HbCS), Hb Icaria, Hb Koya Dora, Hb Seal Rock and Hb Paksé (middle East, Mediterranean and South East Asia) [75-79] and structural mutations causing highly unstable α-globin variants; for example, Hb Quong Sze, Hb Suan Dok, Hb Petah Tikvah, Hb Adana, Hb Aghia Sophia [54,80-84]. These common mutations are summarised in Tables 1 and 2. A regularly updated overview is provided by the HbVar web-site [85].

**Table 1:** Non-deletional mutations that cause α-thalassaemia

Affected sequence	Affected gene *	Mutation(s)	HGVS	Synonym Hb- name	Distribution	Phenotype
**mRNA processing**						

Cryptic splicing	α2	Cd22 C>T	c.69C>T p.Gly23Gly		Surinamese	α^+^
IVS(donor)	α2	IVS I(-5 nt)	c.95+2_95+6delTGAGG		Mediterranean	α^+^
	α1	IVS I-1(g>a)	c.95+1G>A		Thai	α^+^
	α2	IVS II-2 (t>a)	c.300+2T>A		North-European	α^+ ^- α^0^
IVS(acceptor)	α2	IVS I-116 (a>g)	c.96-2A>G		Dutch	α^+^
	α1	IVS I-117 (g>a)	c.96-1G>A		Asian Indian	α^+^
	α2	IVS II-142 (g>a)	c.301-1G>A		Argentinian	α^+ ^- α^0^
	α1	IVS II-148 (a>g)	c.301-2A>G		Iranian	α^+^
Poly A signal	α2	PA del 16 bp	c.*74_*89delCCTTCCTGGTCTTTGA		Arab	α^+ ^- α^0^
	α2	PA1 (AATAAG)	c.*94A>G		Middle East, Med	α^+ ^- α^0^
	α2	PA2 (AATGAA)	c.*92A>G		Med, Chinese	α^+ ^- α^0^
	α2	PA3 (AATA- -)	c.*93_*94delAA		Asian Indian	α^+ ^- α^0^
	α2	PA4 (AATAAC)	c.*94A>C			α^+ ^- α^0^
**mRNA translation**						

Initiation codon	- α^3.7^	init ATG>GTG	c.1A>G p.Met1Val		African	α^0^
	- α^3.7 II^	init (-2 bp)	c.-2_-3delAC		N-African, Med	α^+ ^- α^0^
	α2	init ATG>ACG	c.2T>C p.Met1Thr		Med	α^+^
	α2	init ATG>A-G	c.2delT p.Met1fs		Vietnam	α^+^
	α1	init ATG>GTG	c.1A>G p.Met1Val		Med	α^+^
	α2	init ATG>-TG	c.1delA p.Met1fs		South-East Asian	α^+^
Exon I	α1	Cd14 G>A	c.44G>A p.Trp15X		Iranian	α^0^
	α2	Cd19 (-G)	c.60delG p.His21fs		Iranian	α^+^
	α2	Cd22 (-C)	c.69delC p.Gly23fs		African	
	α2	Cd23 (G>T)	c.70G>T p.Glu24X		Tunesian	α^0^
	- α	Cd30/31(-2 bp)	c.94_95delAG		African	α^0^
Exon II	α2	Cd39/41(del/ins)	c.118_126delACCAAGACC dup TACTTCCC p.Thr40fs		Yemenite-Jewish	α^+^
	α1	Cd51-55(-13 bp)	c.155_167delGCTCTGCCCAGGT p.Gly52fs		Spanish	α^+^
	α1	Cd62(-G)	c.187delG p.Val63fs		African	
	α1	Cd78(-C)	c.237delC p.Asn79fs		Black/Chinese	
	α2	Cd90 A>T	c.271A>T p.Lys91X		Middle Eastern	α^+^
Exon III	α1	Cd108(-C)	c.326delC p.Thr109fs		Jewish	α^+ ^- α^0^
	α2	Cd113/114(-C)	c.342_343delC p.Leu114fs		Unknown	
	α2	Cd113-116(-12 bp)	c.[339C>G;340_351delCTCCCCGCCGAG]	Leida	Spanish	α^+ ^- α^0^
	α2	Cd116 G>T	c.349G>T p.Glu117X		African	α^+^
	α1	Cd131(+T)	c.396_397insT	Pak Num Po	Thai	α^0^
Termination codon	α2	Term Cd TAA>CAA	c.427T>C p.X143Gln	Constant Spring	South-East Asian	α^+^
	α2	Term Cd TAA>AAA	c.427T>A p.X143Lys	Icaria	Med	α^+^
	α2	Term Cd TAA>TCA	c.428A>C p.X143Ser	Koya Dora	Indian	α^+^
	α2	Term Cd TAA>GAA	c.427T>G p.X143Glu	Seal Rock	African	α^+^
	α2	Term Cd TAA>TAT	c.429A>T p.X143Leu	Paksé	Laotian, Thai	α^+^
**Post translational**						

Exon I	- α	Cd14 T>G	c.43T>G p.Trp15Gly	Evanston	African	α^+^
	α2	Cd21 G>T	c.64G>T p.Ala22Ser	Zoetermeer	Dutch	α^+^
	α2	Cd21 G>C	c.64G>C p.Ala22Pro	Fontaine-bleau	French	α^+^
	α2	Cd29 T>C	c.89T>C p.Leu30Pro	Agrinio	Med	α^+^
	α2	Cd30(-3 bp)	c.91_93delGAG p.Glu31del		Chinese	α^+ ^- α^0^
	α2	Cd31 G>A	c.95G>A		Chinese	α^+ ^- α^0^
Exon II	α2	Cd32 G>A	c.99G>A p.Met33Ile	Amsterdam	Surinamese black	α^+ ^- α^0^
	α2	Cd33 T>C	c.101T>C p.Phe34Ser	Chartres	French	α^+^
	α2	Cd35 T>C	c.106T>C p.Ser36Pro	Evora	Filipino, Portugese	α^+ ^- α^0^
	α1	Cd37(-3 bp)	c.112_114delCCC	Heraklion	Greece	α^+ ^- α^0^
	α2	Cd59 G>A	c.179G>A p.Gly60Asp	Adana	Chinese	α^+ ^- α^0^
	α1	Cd60/61(-3 bp)	c.184_186delAAG	Clinic	Spanish	α^+ ^- α^0^
	α2	Cd62(-3 bp)	c.187_189delGTG	Aghia Sophia	Greek	α^0^
	α1	Cd64-74(-33 bp)	c.196_228delGCGCTGACCAAGGCCGTGGCGCACGTGGAC		Greek	α^0^
	α2	Cd66 T>C	c.200T>C p.Leu67Pro	Dartmouth	Caucasian	α^+ ^- α^0^
	α2	Cd93 T>G	c.281T>G p.Val94Gly	Bronte	Italian	α^+^
	α1	Cd93-99(dup21 bp)	c.280_300dupGTGGACCCGGTCAACTTCAAG		Iranian	α^+ ^- α^0^
Exon III	α2	Cd103 A>T	c.311A>T p.His104Leu	Bronovo	Turkish	α^+^
	α2	Cd104 G>A	c.314G>A Cys105Tyr	Sallanches	French/Pakistani	α^+^
	α1	Cd104 T>A	c.313T>A p.Cys105Ser	Oegstgeest	Surinamese	α^+^
	α2	Cd108 C>A	c.326C>A p.Thr109Asn	Bleuland	Surinamese	α^+^
	α2	Cd109 T>G	c.329T>G p.Leu110Arg	Suan Dok	Thai	α^+^
	α	Cd110 C>A	c.332C>A Ala111Asp	Petah Tikva	Middle East	α^+^
	α1	Cd119 C>T	c.358C>T p.Pro120Ser	Groene Hart or Bernalda	Moroccan	α^+^
	α2	Cd125 T>G	c.377T>G p.Leu126Arg	Plasencia	Spanish	α^+^
	α2	Cd125 T>C	c.377T>C p.Leu126Pro	Quong Sze	Chinese	α^+^
	- α^3.7^	Cd125 T>A	c.377T>A p.Leu126Gln	Westeinde	Jewish	α^0^
	α1	Cd129 T>C	c.389T>C p.Leu130Pro	Tunis-Bizerte	Tunisian	α^+^
	α2	Cd129 T>C	c.389T>C p.Leu130Pro	Utrecht	Dutch	α^+^
	α2	Cd130 G>C	c.391G>C p.Ala131Pro	Sun Prairie	Asian Indian	α^+^
	α2	Cd131 T>C	c.394T>C p.Ser132Pro	Questembert	French/Yugoslavian	α^+^
	α2	Cd132 T>G	c.398T>G p.Val133Gly	Caen	Caucasian	α^+^
	α2	Cd136 T>C	c.410T>C p.Leu137Pro	Bibba	Caucasian	α^+^
						

Del; deletion, Dup; duplication, ins; insertion, Cd; codon, PA; poly(A)signal, term; termination codon, init; initiation codon

* The duplicated α-globin genes, α_1 _and α_2 _on the short arm of chromosome 16, are named HBA1 and HBA2 respectively according to the HUGO nomenclature. For practical reasons there will be referred to them as α-genes in the text.

**Table 2:** Alpha-thalassaemia mutations in different ethnic groups

Ethnic group	Type of thal	Mutation(s)	Occurrence
Mediterranean	α^0^	- - ^MED I^	Relatively frequent in Greece, Cyprus, Turkey
		- - ^MED II^	Relatively rare, Southern Italy, Greece, Turkey
		- (α)^20.5^	Common in Greece, Cyprus, Turkey
	α^+^	- α^3.7^	Common in all Mediterranean populations
		α ^IVS I(-5 nt) ^α	Relatively common
		α^Constant Spring ^α	Relatively rare in Greece, independent event from CS in SE-Asia
		αα ^cd119C>T^	Hb Groene Hart, common in Moroccan, Tunisian
	α^+ ^- α^0^	α ^PA1(AATAAG) ^α	In homozygous causing HbH disease, compound heterozygote with α^0 ^-thal deletion causing an Hb Bart's HF-like syndrome
		α ^PA2(AATGAA) ^α	
			
Middle East	α^0^	- - ^MED I^	Common in Iran, Palestinians, Arab population
	α^+^	- α^3.7^	Common in Iran, Palestinians, Arab population
	α^+ ^- α^0^	α ^PA1(AATAAG) ^α	Relatively common in Arab population
			
India	α^+^	- α^3.7^	Common
		- α^4.2^	Less common
		α ^Koya Dora ^α	Relatively rare
		α ^IVS I-117 ^α	Relatively rare
	α^+ ^- α^0^	α ^PA3(AATA- -) ^α	Also found in Hindustani from Surinam
			
South-East Asia	α^0^	- - ^SEA^	Most common deletion among Asians world wide
		- - ^FIL^	Mainly in Philippinians
		- -^THAI^	Common among Thai
	α^+^	- α^3.7^	Relatively common
		- α^4.2^	Relatively rare
		α^Constant Spring ^α	One of the most common non-deletion variants in Chinese
		α^Suan Dok ^α	Highly unstable α-chain
		α^Quong Sze ^α	Highly unstable α-chain
		α^Paksé ^α	Highly unstable α-chain, found in Thai, Laotian
		α^init A-G ^α	Common in Vietnam
		α^init -TG ^α	Common in South-East Asia
			
African, Afro-American and Afro-Caribbean	α^0^	- α^3.7 init GTG^	One of the few α^0^-thal alleles in African population
		- α^3.7 init (-2 bp)^	One of the few α^0^-thal alleles in North-African population
	α^+^	- α^3.7^	Common
		- α^3.7 Cd14 T>G^	Hb Evanston, relatively rare, also found as α^T^α allele in Surinamese
		α^Seal Rock ^α	Relatively rare
			
North-European, Caucasian	α^0^	- - ^Dutch I^	Rare among Dutch, Germans
		- - ^Dutch II^	Rare, found in different Dutch families with common ancestor
		- - ^Brit^	Rare, found in different British families with common ancestor
	α^+^	α^IVS1-116^α	Rare, found in different independent Dutch families
		α^IVSII-2^α	Very rare, found in Dutch families with common ancestry
		α^cd129^α	Hb Utrecht, found occasionally in Dutch families
			

Adapted from Barbara J. Bain, Haemoglobinopathy Diagnosis 2^nd ^edition 2006 {Bain, 2006 126/id}

### α^0^-thalassaemia due to deletions

**Figure 6:**
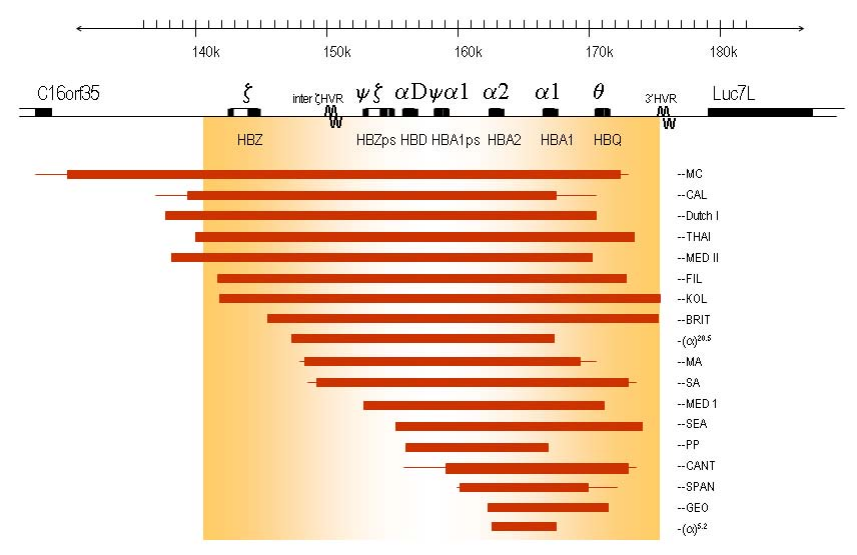
**Deletions of two α-genes giving rise to α^0^-thalassaemia**.

**Figure 7:**
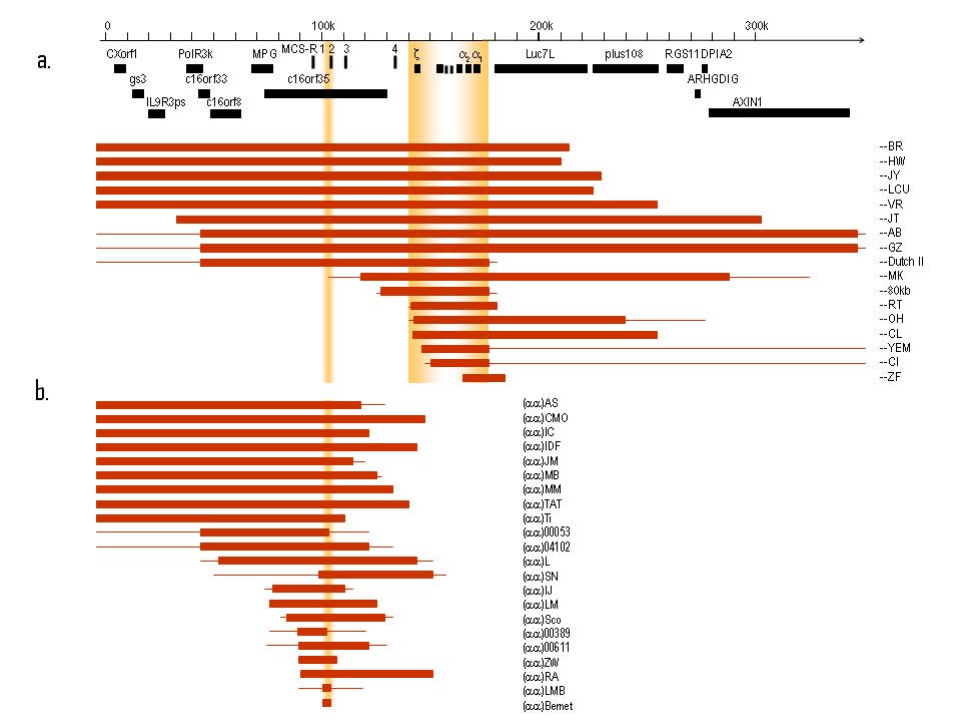
**(continuation of figure 6) **a**. Large deletions involving both α-genes and **b**. deletions of the α-globin regulatory region leaving the α-genes intact**.

The complete or partial deletion of both α-genes in *cis *results in no α-chain synthesis directed by these chromosomes *in vivo *(Figures 6 and 7a). Homozygotes for such deletions have the Hb Bart's Hydrops Foetalis Syndrome. Many deletions were described which remove the ζ- and α-genes and although heterozygotes appear to develop normally, it is unlikely that homozygotes could survive even the early stages of gestation since neither embryonic (ζ_2_γ_2_) nor foetal (α_2_γ_2_) haemoglobins could be made. Rare deletions causing α^0^-thalassaemia remove the regulatory region, which lies 40-50 kb upstream of the α-globin gene cluster leaving the α-genes intact. This region composed of four multispecies conserved sequences (MCS), called MCS-R1 to R4, correspond to the previously identified erythroid-specific DNAse1 hypersensitive sites referred to as HS-48, HS-40, HS-33 and HS-10. Of these elements, only MCS-R2 (HS-40), 40 kb upstream from the ζ globin mRNA cap-site has been shown to be essential for α globin expression. An overview showing all currently known (αα)^T ^deletions is given in Figure 7b, a regularly updated summary is given elsewhere (deletions are reviewed in detail in Higgs et al Disorders of Hemoglobin Cambridge University Press 2009 [69].

A different spectrum of both α^+^- and α^0^-thalassaemia mutations is often found in different populations as indicated in Table 2 {Bain, 2006 126/id}. Ethnic origin may therefore guide molecular diagnosis. Knowledge of the mutations found in a specific population may allow strategic choice in laboratory diagnostics, especially in selection of the molecular techniques to be applied.

## Genotype/Phenotype Correlations

Although there are now ~128 different molecular defects known to cause α thalassaemia and an ever increasing number of potential interactions, the clinical phenotypes (broadly classified as α thalassaemia trait, HbH disease and Hb Bart's hydrops foetalis) resulting from the interactions between these various molecular defects can be simply summarised as in Table 3. The severity of the clinical phenotype correlates very well with the degree of α globin chain deficiency. An important additional point is that, in general, interactions involving non-deletional forms of α^+^-thalassaemia result in a more severe phenotype than in those with deletional forms of α^+^-thalassaemia [69,87-100].

**Table 3:** Interactions in α-thalassaemia

		α^+^					α^0^	
		**α α**	**α α^T^**	**- α**	**α^T^α**	**- α^T^**	**- -**	**(αα)**

α^0^	- -	T	H	H	H, Hy	H	Hy	

	(αα)	T		H				

								

α^+^	- α^T^	T	Unk	H	T	H		

	α^T^α	T	Unk	T	T, H			

	- α	T	Unk	T				

	α α^T^	T	Unk					

	α α	N						

Abbreviations: (αα), non-deletion α^0 ^thalassaemia (due to upstream deletion); --, deletion α^0 ^thalassaemia;

N, non-thalassaemic; T, α-thalassaemia trait; H, HbH disease; Hy, Bart's hydrops foetalis syndrome; Unk, unknown, not observed yet. The severity of the condition, thalassaemia trait or HbH disease is determined by the severity of down-regulation by the non-deletion mutant, this remains to be determined through further observation (adapted from Weatherall and Clegg 2001)[5].

## Diagnosis and diagnostic methods

Initial laboratory testing should include a complete blood count with red cell indices, HPLC or Hb electrophoresis and eventually α/β-globin chain synthesis ratio measurement. The latter procedure, however, is sometimes bypassed by DNA analysis as a less complicated method to diagnose α-thalassaemia.

### Haematology

**Figure 8:**
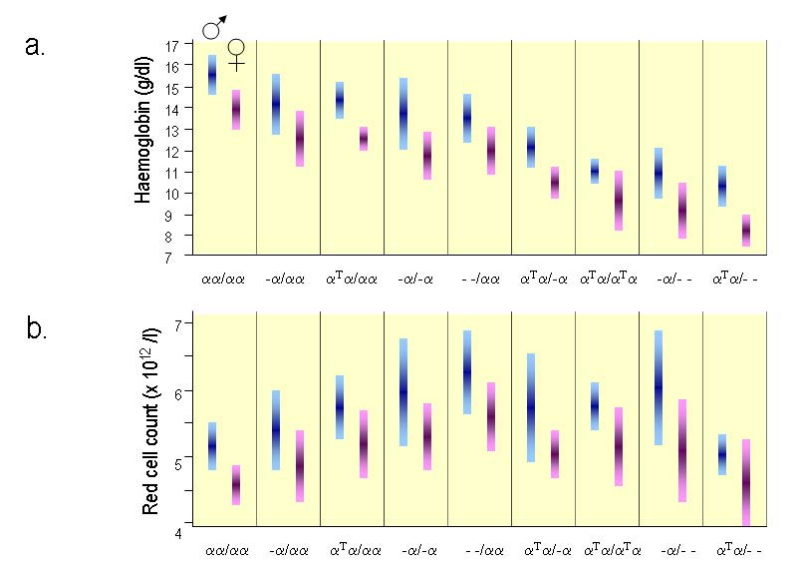
**Red blood cell indices in patients with various genotypes associated with α-thalassaemia**. The bar shows the mean and standard deviation. **a**. Haemoglobin level (Hb in g/dl), **b**. Red Cell Count (RBC indicated as × 10^12^/l), these are sex-dependent (blue for male distribution, pink female distribution). (adapted from Higgs 1993, Wilkie 1991) [44,101].

**Figure 9:**
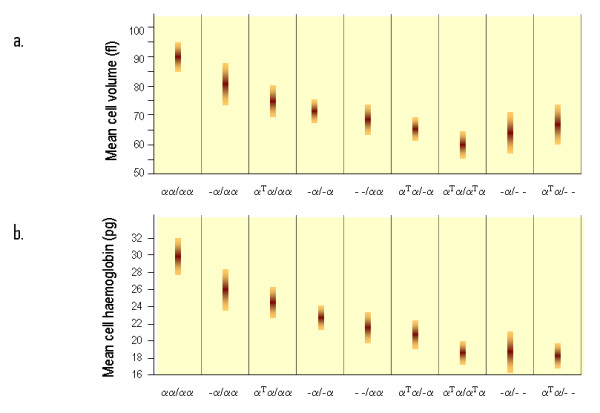
**(continuation of figure 8) **a**. Mean Cellular Volume (MCV in fl) and **b**. Mean Cell Haemoglobin (MCH in pg) (adapted from Higgs 1993, Wilkie 1991) **[44,101].

**Figure 10:**
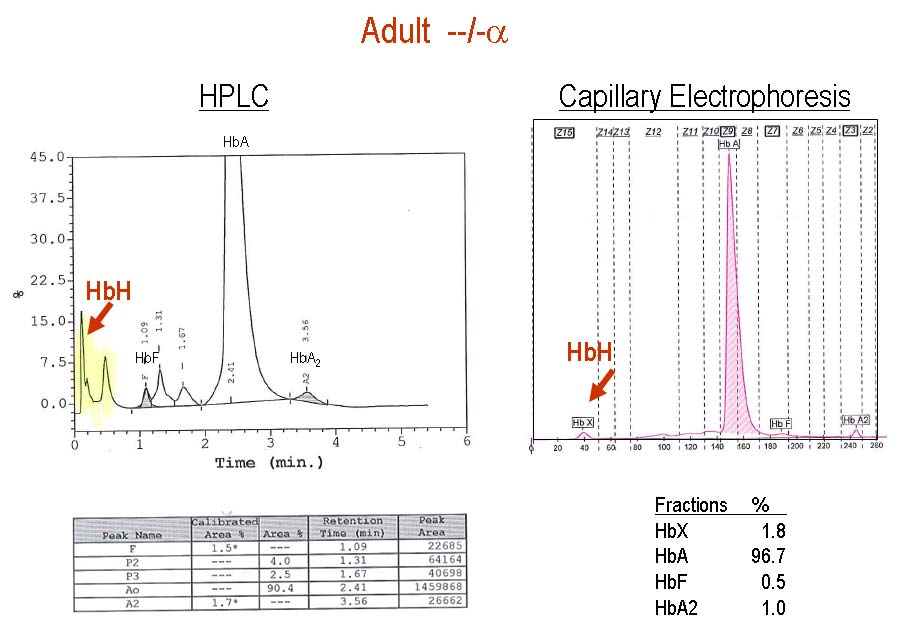
**HPLC and Capillary Hb electrophoresis patterns of an adult with HbH disease**. The HbH (β4 tetramers) peak elutes from the column as a compressed fraction, and as a fast moving fraction in electrophoresis.

**Figure 11:**
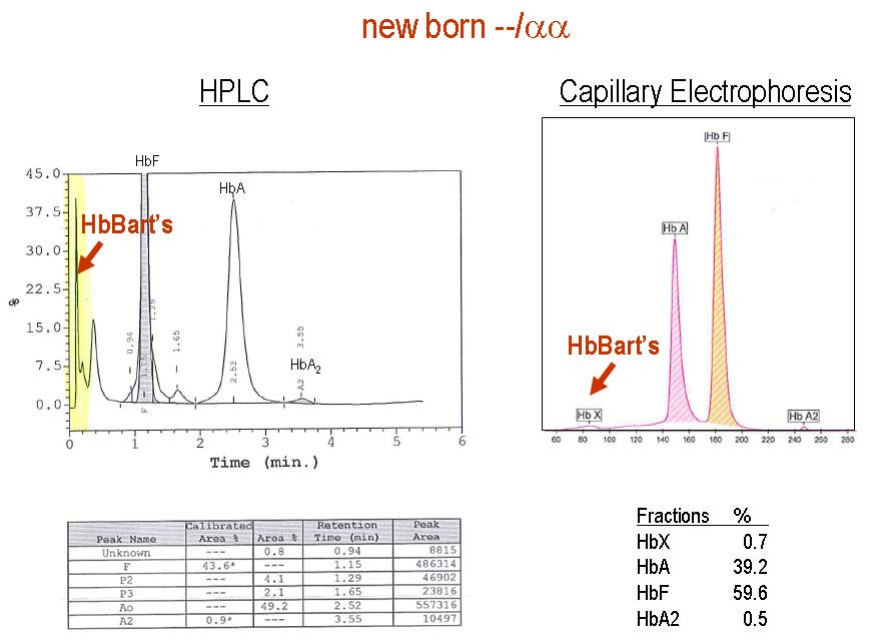
**HPLC and Capillary Hb electrophoresis patterns of a neonate with α thalassaemia trait (--/αα) and a significant amount of Hb Bart's (γ4 tetramers)**. Hb Bart's in newborns with α thalassaemia disappears rapidly after birth. In newborns with Hb H disease, Hb Bart's will be substituted by HbH after birth. In Hb Bart's hydrops foetalis syndrome due to homozygosity of α^0^-thalassaemia only Hb Bart's is seen.

**Figure 12:**
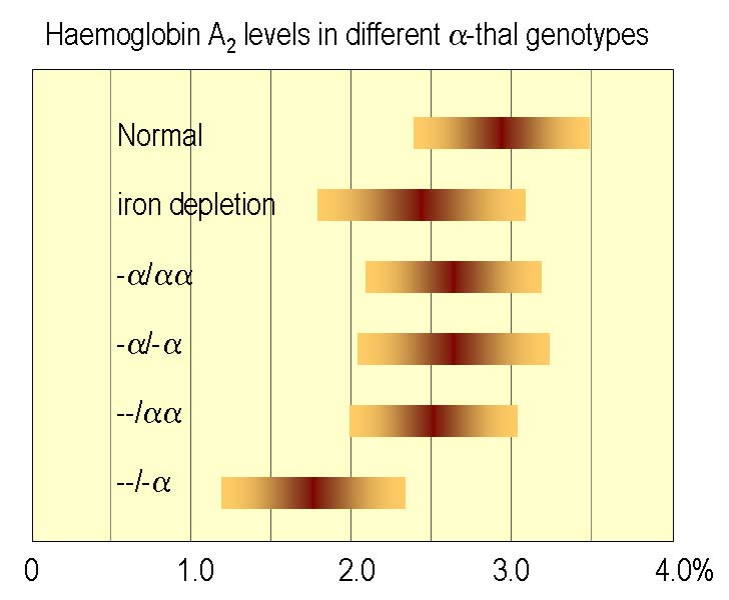
**Mean and standard deviation of HbA_2 _in different α-thalassaemia genotypes**.

**Figure 13:**
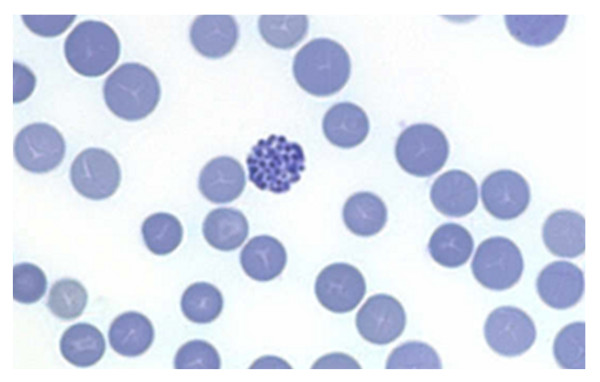
**An inclusion body positive cell seen in Brilliant Cresyl Blue stained red cells of a α^0^-thalassaemia carrier**. Inclusion Bodies are β4-tetramers precipitating on the red cell membrane, which damages the membrane and induces haemolysis. HbH is unstable and inclusion body positive cells are more difficult to find in older blood samples. The number of inclusion body cells seen after staining is much lower in α^0^-thalassaemia carriers than in patients with HbH disease (1 in 5-10 fields versus several per field at 1000× microscopic magnification).

The red blood cell indices in patients with various genotypes associated with α-thalassaemia are depicted in figure 8 and 9. In general, the degree of microcytic (low MCV), hypochromic (low MCH) anaemia (low Hb) depends roughly on the number of α genes mutated and correlates well with the reduction in α-chain synthesis predicted for each mutant [5,44,101]. The combined use of HPLC and Capillary Electrophoresis to separate abnormal haemoglobin fractions is of particular importance to demonstrate HbH in individuals with HbH disease (figure 10) and Hb Bart's in newborns carrying α-thalassaemia determinants or any Hb variant associated with an α-thalassaemia phenotype (figure 11). Hb Bart's is found in a large proportion of neonates with α-thalassaemia but does not detect all cases with mild α-^3.7^/αα interactions and does not clearly distinguish the various α thalassaemia genotypes [11,19,102]. A reduction in HbA_2 _level is sometimes indicative of α-thalassaemia trait. Although this nicely distinguishes α and β thalassaemia trait it can hardly be relied upon as a guide to the degree or type of α thalassaemia. A reduction in the level of HbA_2 _is only distinctive in patients with HbH disease (see figure 12)[103]. Staining the peripheral blood cells with 1% Brilliant Cresyl Blue is a sensitive method to visualise inclusion bodies in the red cells. The typical inclusion-body cells have a golf-ball like appearance with stippling regularly distributed over a blue stained background (Figure 13). They appear occasionally (one to two cells in approximately 10 fields 1000× magnification) in carriers of the --/αα genotype and in carriers of many nondeletional defects. Numerous red cells containing inclusions can be seen in the BCB-stained peripheral blood smears of patients with HbH disease.

### Alpha/beta-globin chain synthesis

Measuring the ratio of α- and β-globin chain synthesis is the most direct approach (at the protein level) to diagnose α-thalassaemia. The procedure was first described by Weatherall and Clegg in 1965 [27] and consists of several steps, including removal of white blood cells, reticulocyte enrichment, *in vitro *globin chain synthesis in the presence of radio-actively labelled Leucine, separation of the newly synthesized, radio-actively labelled α- and β-globin chains and measurement of the radio-active signal. If the α/β ratio appears lower than ~0.8 this is indicative of α-thalassaemia, a ratio around 0.75 being consistent with the loss of expression of a single α gene (-α/αα), 0.5 for two α genes (--/αα) and 0.25 for three α-genes (--/-α) [24,25,27,28].

### Molecular analysis

Over the past 30 years it has become increasingly possible to diagnose α thalassaemia accurately and define the precise defects underlying these disorders using a variety of molecular genetic approaches. Ultimately, most α globin rearrangements have been characterised by Southern blotting and DNA sequence analysis. However, for today's diagnostic demands these techniques are far too laborious to apply in each case, and from the original work defining these mutations, rapid screening assays have been developed.

Gap-PCR has been developed for the 7 most common α-thalassaemia deletions. This method is applied to detect the 2 most common α^+ ^thalassaemia deletions -α^3.7 ^and α-^4.2 ^and the 5 α^0^-thalassaemia deletions -(α)^20.5^, - - ^SEA^, - - ^Med I^, - - ^Thai ^and - - ^Fil ^[104-106].

When a point mutation (non-deletional mutation) is suspected re-sequencing the α genes has become a routine procedure. The α genes are relatively small (~1.2 kb) which allows them to be sequenced rather easily compared to many other genes involved in human genetic disease, like for instance Duchenne Muscular Dystrophy (DMD gene; ~2.3 Mb), Cystic Fibrosis (CF-gene; ~250 kb) and Breast Cancer (BRCA1 and BRCA2 genes, ~16 and ~10 kb respectively)[107-109]. However, the GC-richness and the high homology between the duplicated α-genes require the use of high fidelity, heat stable polymerases, specific reaction conditions (using DMSO and betaine) and limits the choice of specific primers for PCR. The α-genes can be conveniently sequenced in two overlapping fragments for each of the duplicated α1 and α2 genes [19,110,111].

**Figure 14:**
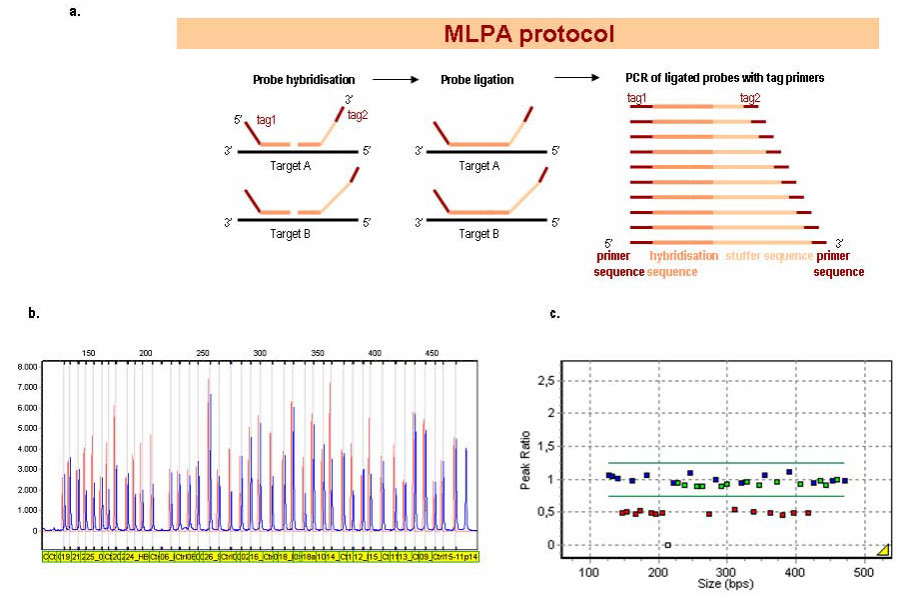
**The principle of Multiplex Ligation dependent Probe Amplification (MLPA)**. **a**. Probe pairs at different locations along the region of interest are hybridised specifically head-to-tail to the target sequence and subsequently ligated. The ligated probes are amplified by quantitative PCR using fluorescent labelled primers complementary to the tag-sequences and separated by capillary electrophoresis on an automated fragment analyzer. **b**. peak heights represent the amount of amplified product of each separate probe pair. **c**. By dividing the peak heights of the patient sample and a normal control for each fragment, the ratio's of 0.5 shown in the graph mark the deletion of certain probes located along the genome, indicating the presence of a deletion of one allele.

For suspected but currently unknown rearrangements, Southern blotting or MLPA analysis may be used. Southern blot is the classical method to detect deletions causing α-thalassaemia [112-117]. More recently Multiplex Ligation-dependent Probe Amplification (MLPA) is used, based on ligation of multiple probe-pairs hybridised across a (usually large) region of interest (Figure 14), followed by semi-quantitative amplification using universal-tag PCR primers and subsequently fragment analysis. This is a valuable alternative for Southern blot analysis and a supplementary method to gap-PCR when investigating known and unknown deletions causing α-thalassaemia [111,118-120].

## Differential diagnosis

Sometimes carriers of α^+^-thalassaemia present with normal haematology, especially carriers of -α^3.7 ^and nondeletional mutations affecting the α1-gene. Such individuals may be normocytic or borderline hypochromic without anaemia. These can only be found by chance during routine molecular analysis for haemoglobinopathies.

Occasionally, especially in countries where thalassaemia is uncommon, α-thalassaemia trait may be confused with iron deficiency anaemia, especially when the iron status is not carefully assessed. Haematological parameters for thalassaemia and iron deficiency are quite similar therefore ferritin levels should be measured. If the microcytic hypochromic parameters persist in a patient with normal levels of ferritin or Zinc Protoporphyrin (ZPP, a measure for long-lasting iron depletion), elevated RBC and normal (or low) HbA_2_, (especially in patients originating from areas where haemoglobinopathies are common) there is a good chance that the individual is a carrier of α-thalassaemia. Molecular analysis is usually required, especially in silent α-thalassaemia and α-thalassaemia trait to confirm the haematological observations.

There is a difference in clinical severity between deletional (most common) and non-deletional HbH disease [43,47,69,89,91,94-96,98,99]. The clinical diagnosis of deletional HbH disease (the mildest form) is often made only after the detection of complications, such as exacerbations of the anaemia induced by infections, growth failure (in children) or findings of splenomegaly [5,43,121,122]. The laboratory findings show a pronounced microcytic hypochromic anaemia and the presence of inclusion bodies. HbH and Hb Bart's are fast moving haemoglobins appearing on electrophoresis or HPLC, however, they are unstable and may go undetected. The more severe forms of HbH disease are predominantly those involving non-deletion mutations, of which Hb CS is the most common in South-East Asia. This form is characterized by a significantly more ineffective erythropoiesis and erythroid apoptosis than the deletion types of HbH disease. The haemoglobin is lower (on average 2 g/dL), but the MCV higher due to overhydration of cells containing HbCS [2,123-127].

Hydrops Foetalis without α-thalassaemia is a common non-specific finding in a wide variety of foetal and maternal disorders [128-131]. The distinguishing features of the Hb Bart's hydrops foetalis syndrome is the presence of Hb Bart's and the total absence of HbF, which is easily differentiated by HPLC or Hb-electrophoresis. Although there have been a few reports of Hydrops Foetalis infants with very low levels of α-chain synthesis and HbH hydrops [45,52,54,55,99,127].

## Genetic counselling and antenatal diagnosis

When both parents carry an α^o ^thalassaemia mutation (--/αα) the risk of their offspring having Hb Bart's hydrops foetalis is 1:4 (25%). When one parent carries α^o ^thalassaemia (--/αα) and the other carries an α^+ ^thalassaemia (-α/αα) the risk of their offspring having HbH disease is 1:4 (25%). If the carrier of α^+ ^thalassaemia is a homozygote clearly the risk of HbH disease is 1:2 (50%). Since there are many different alleles of α^o ^and α^+ ^thalassaemia, genetic counselling may be more complex than outlined in this simple model.

In families with α thalassaemia the main reason for offering prenatal diagnosis is to avoid pregnancies with the Hb Bart's hydrops foetalis syndrome which causes neonatal death. Continued pregnancy may also present a considerable risk to the mother. Prenatal diagnosis for Hb Bart's is offered when both parents are found to be carriers of α^0^-thalassaemia trait. This is of most importance in individuals of South East Asian origins. Although some reports have demonstrated the feasibility of treating this syndrome, the lack of knowledge of the long-term prognosis and the capacity for treating such individuals probably do not justify changing the conventional management of offering prenatal diagnosis and selective abortion for Hb Bart's Hydrops Foetalis syndrome.

The syndrome of HbH disease is usually mild (thalassaemia intermedia) but there is considerable variability in the clinical and haematological severity. Although, precise characterisation of the mutations involved allows some prediction of the severity of the disease this is by no means certain, which makes prenatal diagnosis offered to parents at risk of having a child with HbH disease a complicated ethical issue. Most cases resulting from simple deletion of the α globin genes are mildly affected. Nearly all severe cases have at least one nondeletional allele. However the clinical course can be influenced by other genetic factors, environmental factors and infections. In rare cases the interaction of α^0^-thalassaemia with a non-deletional α^+^-thalassaemia allele has led to individuals with hydrops foetalis syndrome [54,99,127]. When there is a risk of such severely affected individuals there may be a case for considering prenatal diagnosis.

## Management including treatment

### Alpha thalassaemia trait

Carriers of α^+^- or α^0^-thalassaemia alleles generally do not need treatment, because their anaemia is either very mild or absent due to a compensating high red blood cell count. On the other hand, once a diagnosis of α thalassaemia trait is made, there is a tendency to discard iron-deficiency as a subsequent cause of anaemia. Carriers of α thalassaemia can be anaemic as a consequence of co-existing nutritional deficiencies, such as iron deficiency, folate or vitamin B12 deficiencies and should be managed correctly from this point of view. Of course prophylactic iron should never be given to carriers of α thalassaemia who are at risk of developing iron overload if treated inappropriately.

### HbH disease

HbH disease may be a mild disorder, but recent studies suggest its clinical course is often more severe than previously recognized [43,122,123,125-127]. As discussed above, the type of mutation influences the clinical severity of HbH disease. The most common form is the deletion type, which causes a milder form of HbH disease. These patients may require intermittent transfusion therapy especially during intercurrent illness. Chronic transfusion therapy is very uncommonly required in this group. However, patients with non-deletional types of HbH disease may have moderately severe splenomegaly and require more regular transfusion and ultimately splenectomy [5,69,125]. In some studies almost half of such patients have required repeated transfusions, particularly in early infancy and later adulthood [2,5,69,124,132]. However, there is a marked clinical variation in both categories. Iron overload is uncommon in HbH disease patients (compared with β thalassaemia) but has been recorded in older patients (>45 years) and those treated with regular blood transfusion.

### Hb Bart's Hydrops Foetalis Syndrome

Most pregnancies in which the foetus is known to have the Bart's hydrops foetalis syndrome are terminated. In a very small number of cases intra-uterine transfusions following early detection of homozygous α^0^-thalassaemia have resulted in the birth of non-hydropic infants, some without significant neurological or congenital abnormalities, however, most survivors experience a stormy perinatal course and a high prevalence of congenital urogenital and limb defects [5,133-137]. Affected infants who survive are good candidates for haematopoietic stem cell transplantation[60,138]. Obstetric complications and the necessity for long-term transfusion therapy are however serious arguments for counselling and selective abortion. Increased risk of both maternal and foetal morbidity should be taken into account when counselling couples at risk for having a child affected with this syndrome [5,44,134].

## Prognosis

There is no reason to think that carriers for α thalassaemia have any altered prognosis for life compared to the normal population. The prognosis for patients with HbH disease who are newly emerging in previously non-endemic countries, like Northern Europe and Northern America, is less clear. Anecdotally many patients with HbH disease appear to lead a normal life in all respects. Some even remain undiagnosed throughout their lives. However, detailed actuarial studies are not available. When complications arise, of course the outcome depends on the awareness and availability of health care systems. Certainly some complications suffered by patients with HbH disease are life threatening in the absence of adequate medical care [5,44,123,124]. A long term problem for all patients with HbH disease is the unwanted accumulation of iron which may be more of a problem for those with severe HbH disease with non-deletional α- thalassaemia [43,139,140].

Clearly, previously undiagnosed and untreated infants with the Hb Bart's hydrops foetalis syndrome die in the perinatal period. The recent attempts to rescue infants with this syndrome either by intra-uterine transfusion or by transfusion in the perinatal period have met with variable success. As discussed above many infants develop other irreversible abnormalities during foetal life and even with rescue the infant will be required, either to receive lifelong blood transfusion and iron chelation therapy, or bone marrow transplantation with its attendant risks.

### Unresolved questions

1. How is the expression of genes in the α- (and β-) globin gene cluster regulated and how can it be influenced? A detailed understanding of globin gene regulation might hold the key to developing new treatments for both α and β thalassaemia.

2. What other factors (genetic and environmental) influence the clinical severity of HbH disease and might explain the large variability even between individuals with the same α globin genotypes?

3. What role does α-thalassaemia play in modifying the natural history of sickle cell disease and β-thalassaemia major? These interactions are not always well understood.

4. In what way are carriers of α-thalassaemia protected from some of the effects of malaria?

## Competing interests

The authors declare that they have no competing interests.

## Authors' contributions

The authors CLH and DRH have contributed equally to the draft of the manuscript. Both authors have read and approved the final manuscript.
